# Refined stratified-worm-burden models that incorporate specific biological features of human and snail hosts provide better estimates of *Schistosoma* diagnosis, transmission, and control

**DOI:** 10.1186/s13071-016-1681-4

**Published:** 2016-08-04

**Authors:** David Gurarie, Charles H. King, Nara Yoon, Emily Li

**Affiliations:** 1 Department of Mathematics, Applied Mathematics and Statistics, Case Western Reserve University, Cleveland, USA; 2 Center for Global Health and Diseases, School of Medicine, Case Western Reserve University, 10900 Euclid Avenue, Cleveland, Ohio USA; 3 Schistosomiasis Consortium for Operational Research and Evaluation, University of Georgia, Athens, Georgia USA

**Keywords:** Mathematical models, Schistosomiasis control, Drug therapy, Disease transmission

## Abstract

**Background:**

*Schistosoma* parasites sustain a complex transmission process that cycles between a definitive human host, two free-swimming larval stages, and an intermediate snail host. Multiple factors modify their transmission and affect their control, including heterogeneity in host populations and environment, the aggregated distribution of human worm burdens, and features of parasite reproduction and host snail biology. Because these factors serve to enhance local transmission, their inclusion is important in attempting accurate quantitative prediction of the outcomes of schistosomiasis control programs. However, their inclusion raises many mathematical and computational challenges. To address these, we have recently developed a tractable stratified worm burden (SWB) model that occupies an intermediate place between simpler deterministic mean worm burden models and the very computationally-intensive, autonomous agent models.

**Methods:**

To refine the accuracy of model predictions, we modified an earlier version of the SWB by incorporating factors representing essential in-host biology (parasite mating, aggregation, density-dependent fecundity, and random egg-release) into demographically structured host communities. We also revised the snail component of the transmission model to reflect a saturable form of human-to-snail transmission. The new model allowed us to realistically simulate overdispersed egg-test results observed in individual-level field data. We further developed a Bayesian-type calibration methodology that accounted for model and data uncertainties.

**Results:**

The new model methodology was applied to multi-year, individual-level field data on *S. haematobium* infections in coastal Kenya. We successfully derived age-specific estimates of worm burden distributions and worm fecundity and crowding functions for children and adults. Estimates from the new SWB model were compared with those from the older, simpler SWB with some substantial differences noted. We validated our new SWB estimates in prediction of drug treatment-based control outcomes for a typical Kenyan community.

**Conclusions:**

The new version of the SWB model provides a better tool to predict the outcomes of ongoing schistosomiasis control programs. It reflects parasite features that augment and perpetuate transmission, while it also readily incorporates differences in diagnostic testing and human sub-population differences in treatment coverage. Once extended to other *Schistosoma* species and transmission environments, it will provide a useful and efficient tool for planning control and elimination strategies.

**Electronic supplementary material:**

The online version of this article (doi:10.1186/s13071-016-1681-4) contains supplementary material, which is available to authorized users.

## Background

Parasitic *Schistosoma* species pose a significant health burden in many developing countries [1]. Broad-based regional schistosomiasis control and local elimination of parasite transmission have been prioritized in the 2012 London Declaration for Neglected Tropical Diseases (http://unitingtocombatntds.org/resource/london-declaration) and the recent World Health Organization 2020 Roadmap on Neglected Tropical Diseases (NTDs) [2].

The parasite has a complex ecology in which it cycles between human and snail hosts through intermediate larval stages, in a manner that strongly embeds transmission within at-risk sub-tropical and tropical ecosystems [3]. A key feature of *Schistosoma* infections (similar to other metazoan macro-parasites) is the highly uneven (heterogeneous) distribution of infection burden among its vertebrate host populations, as evident in both experimental and field data [4–6]. Other heterogeneous factors that play important roles in perpetuation of schistosomiasis include variations in local human demographics and exposure frequencies, patchy transmission habitats, and the seasonality of rainfall and temperature factors that affect snail abundance [7].

Modeling of such systems is a challenging task, but to provide more accurate estimates for current control programs, newer models should account for the influential features of in-host biology, transmission environment, diagnostic uncertainties, and the potential efficiency of different control interventions. Conventional approaches based on mean worm burden (MWB) formulations [4, 8–11] have had several shortcomings in this respect. They have used ad-hoc assumptions about worm load distributions and appear to have oversimplified some components of the transmission system. As a result, infection rates in such systems and the modeled impacts of treatment tend to be overestimated [12]. Individual-based modeling approaches (e.g. [13]) could potentially address some of these issues, but individual-based models have significant limitations in terms of accessibility and programming requirements, particularly for large populations.

The stratified worm burden (SWB) approach occupies an intermediate place between MWB and individual-based models, and offers many advantages [12, 14]. Among other features, it provides a natural way to account for worm dispersion in demographically-structured host populations. Our earlier work with SWB [14, 15] was limited in terms of within-host parasite biology, as we had assumed perfect mating and uniform egg-release by all worm male-female pairs, independent of accumulated burden. Here, we refine the earlier SWB model to account for influential components of in-host biology including the aggregated distribution of worm burden, worm mating probabilities, density-dependent worm fecundity, and random features of egg-release [12, 16]. These newly incorporated biological parameters allow us more realistic simulation of diagnostic egg-tests and of environmental egg release and its effect on the force of infection to intermediate host snails. We have developed a new Bayesian calibration procedure that recognizes many uncertainties of modeling infection data based on standard egg-count tests [17–22]. The goal of this calibration approach was to identify the most ‘likely’ model parameter choices consistent with the collected field data. The posterior parameter ensembles for a community could then be used for dynamic simulations of control interventions, incorporating the uncertainties about diagnosis and transmission reflected in the input data. The model/data uncertainties thus yield more robust statistical estimates, including credible ranges for the projected treatment outcomes.

In the current paper, we explain how we have applied the new model and calibration methodology to the field data collected in survey and control studies of *S. haematobium* in coastal Kenya [23–26]. These data provide a fairly complete demographic coverage of several communities, well-suited for our analysis. Because we had also used this data set in our previous modelling using a simpler SWB model (i.e. without in-host biology [14, 15]), we can compare the two models to demonstrate how inclusion of these biological factors can result in more accurate projections of post-treatment infection outcomes.

## Methods

### Description of stratified worm burden system with in-host biology

In the SWB approach [12, 14], the dynamic variables included in the modeling framework are host population strata {*h*_*k*_(*t*)}, (Table 1) which are defined by their actual worm burden values. In our formulation, strata {*h*_*k*_} and transition rates between them are determined by a fixed worm count increment *Δw* - (Table 2). We think of Δ*w* as mating threshold, so that hosts carrying less than Δ*w* worms (stratum *h*_0_) are considered non-infective.

**Table 1:** System variables in the SWB model

System variable	Symbols
SWB prevalence strata:	
*k Δw* ≤ *w* < (*k* + 1)*Δw* with worm burden increment Δ*w*	$$ \begin{array}{c}\hfill \left\{{h}_k(t):k=0,1,\dots \right\}\hfill \\ {}\hfill {\displaystyle {\sum}_k{h}_k=1}\hfill \end{array} $$
Demographically structured SWB:	
Child	*h*_*k*_^*C*^(*t*)
Adult	*h*_*k*_^*A*^(*t*)
Population densities per unit habitat:	
Human	*H* (*t*)
Snail (susceptible, infected, patent)	*N*(*t*) = *x*(*t*) + *y*(*t*) + *z*(*t*)

**Table 2:** SWB system

Host population *H* = ∑_* k* = 0_^*n*^ *h*_*k*_ is divided into burden strata {*h*_*k*_} by their worm load (*w* = # adult worms): *k Δw* ≤ *w* < (*k* + 1)*Δw* for *h*_*k*_. The partition is determined by worm-step *Δw* ≥ 1, that serves as hypothetical mating threshold. So *h*_0_ are infection-free (no mated couples), while for *h*_*k*_ (*k* ≥ 1) its mated count (expected number of couples) given by function (Eq. 1). The transitions among strata $$ \begin{array}{cccccc}\hfill \downarrow {S}_0\hfill & \hfill \hfill & \hfill \downarrow {S}_1\hfill & \hfill \hfill & \hfill \hfill & \hfill \downarrow {S}_n\hfill \\ {}\hfill {h}_0(t)\hfill & \hfill \underset{\gamma_1}{\overset{\lambda }{\rightleftharpoons }}\hfill & \hfill {h}_1(t)\hfill & \hfill \underset{\gamma_2}{\overset{\lambda }{\rightleftharpoons }}\dots \hfill & \hfill \underset{\gamma_n}{\overset{\lambda }{\rightleftharpoons }}\hfill & \hfill {h}_n(t)\hfill \\ {}\hfill \downarrow \mu \hfill & \hfill \hfill & \hfill \downarrow \mu \hfill & \hfill \hfill & \hfill \hfill & \hfill \downarrow \mu \hfill \end{array} $$ are determined by force of infection (FOI) *λ* (= rate of worm accumulation/Δ*w*), resolution rates *γ*_*k*_ = *k γ* (*γ* - mean worm mortality), and population turnover rate *μ* (mortality, maturation, migration, etc.). Source terms *S*_*k*_ represent demographic inputs from related population groups, e.g. for children *S*_0_ = *b*_*H*_ (birth rate), with *S*_*k* ≥ 1_ = 0 (as all newborns are infection-free); whereas adult sources come from maturing child strata. For the interested reader, a version of the SWB model programmed in Mathematica software is provided as Additional file 1	

Because low worm burden is very difficult to measure in living humans, there are no accurate estimates of the relevant minimal Δ*w*. Theoretical arguments suggest relatively low values, Δ*w = 5* [9, 14]. Experimental data from primate infections [27] predicts Δ*w* ≈ 40. Currently, we use an intermediate value of Δ*w* = 10 in our numeric implementation of the SWB system.

**Fig. 1:**
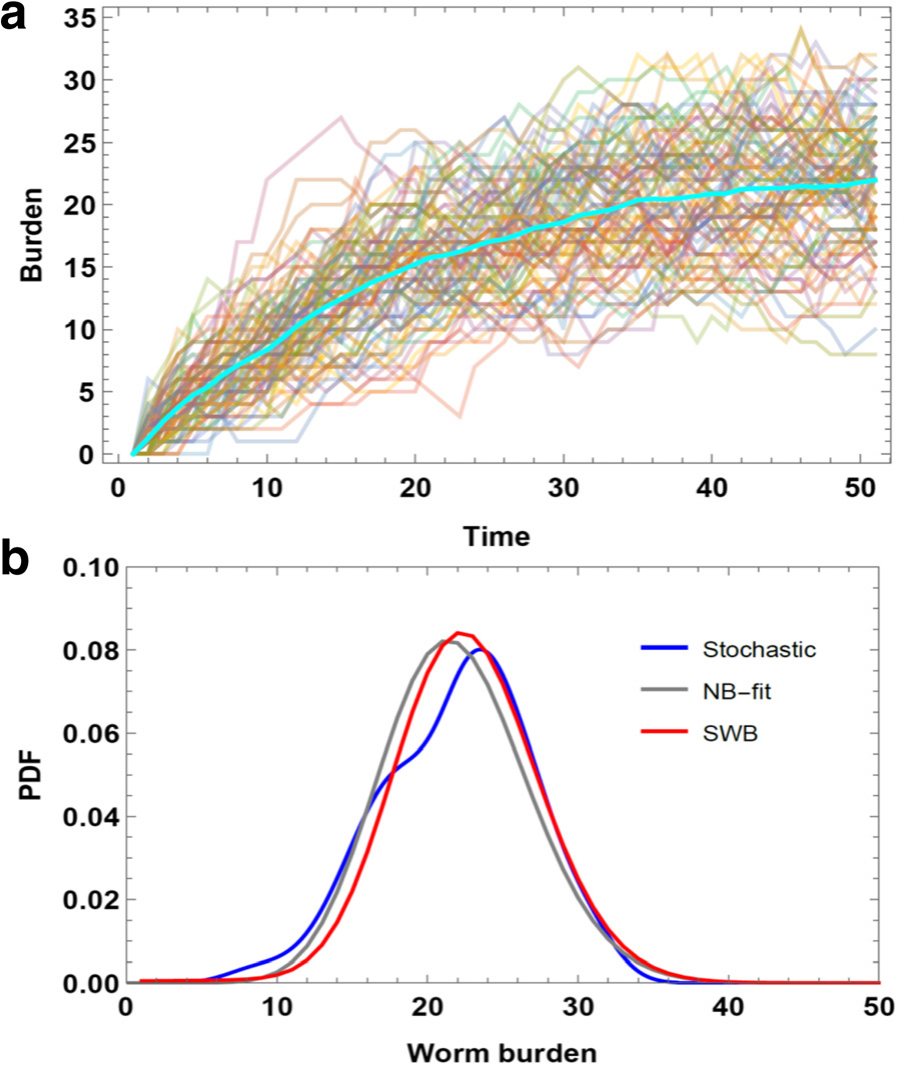
Comparison of equilibrium worm burden distributions. The SWB distribution {*h*_*k*_} of *Schistosoma* worm burden can be viewed as probability distribution function (PDF) representing an ensemble of stochastic agents (human hosts) having a prescribed mean rate of worm accumulation *λ*Δ*w* and worm resolution (death) rate *γ*, yielding an equilibrium level of infection over time. In Panel **a**, we used stochastic individual-agent simulation to repeatedly follow an ensemble of 200 hosts with prescribed mean *λ*,*γ*, to determine their progression from no infection to an equilibrium endemic state. The graph shows the multiple ensemble histories and their mean (*thick line*) which closely follows relaxation dynamics of earlier deterministic models [8], i.e. $\frac{dw}{dt}=\lambda -\gamma w$, approaching equilibrium w* = *λ*/*γ*. In Panel **b**, the PDF of stochastic simulation equilibrium values (*blue line*) is compared to a fitted negative binomial curve, *NB*(*k*, w*) (*gray line*) and to an ensemble of equilibrium SWB model predictions {*h*_*k*_(*λ*/*γ*)} (*red line*). We observe close proximity of the three curves, justifying the view that SWB approximates a stochastic agent model in terms of ensemble PDF, given identical *λ*,*γ*. The resulting worm distribution patterns are highly aggregated (*k* = 231 for fitted NB) and close to a Poisson distribution, in contrast to the highly overdispersed patterns seen for patient egg-count data [16]

A single SWB system describes a homogeneous population, determined by human FOI *λ* (rate of worm accumulation), worm mortality *γ*, and host turnover (demographic) rate, *μ*. Dynamic variables {*h*_*k*_(*t*)} obey coupled differential equations with matrix *A*(*λ*, *μ*, *γ*) and sources {*S*_*k*_(*t*)}. The latter account for demographic changes to SWB populations (birth, death, maturation, migration; for details see Additional file 2 and [12, 14]). In some applications (e.g. model calibration), we ask for equilibrium solutions for SWB systems to align with endemic infection levels. For a single SWB system, equilibrium distribution ĥ* = {*h*_k_^*^(*λ*/*γ*, *μ*/*γ*)} depends on two dimensionless parameters (*λ*/*γ*, *μ*/*γ*) (Additional file 2). For *μ* = 0 (no population turnover), the sequence {*h*_k_^*^} becomes a Poisson distribution with mean $\overline{w}=\lambda /\gamma$, which is equivalent to the equilibrium worm burden (MWB) of a MacDonald-type system [8]. For typical demographic turnover (small *μ*/*γ* ≪ 1), distribution {*h*_k_^*^} values are close to Poisson or negative binomial, with high aggregation (see Fig. 1 and [12]).

### Random egg-release by hosts and SWB communities: simulation of egg-test data

In the updated SWB, two factors determine egg-accumulation by human hosts: the number of fertilized females (mated worm pair count), *ϕ*, and the worm fecundity factor, *ρ*. Both depend on worm burden *w*, and can be estimated as functions of *w*, or for SWB models, the stratum number, *k*. The mated worm count depends on worm mating patterns and worm accumulation in hosts [12, 28]. Some commonly used assumptions about mating, i.e. random worm acquisition and monogamous mating, yield a binomially-distributed sex ratio in each *w*-stratum (*w* adult worms), hence the mated count,

1 $$ \phi (w)=\frac{w}{2}\left[1-{2}^{-w}\left(\begin{array}{c}\hfill \hfill \\ {}\hfill w\hfill \end{array}\begin{array}{c}\hfill w\hfill \\ {}\hfill /\hfill \end{array}\begin{array}{c}\hfill \hfill \\ {}\hfill 2\hfill \end{array}\right)\right];\kern1em \mathrm{or}\kern0.62em {\phi}_{\mathrm{k}}=\phi \left(k\varDelta w\right) $$

for the *h*_*k*_-stratum. Worm fecundity *ρ*(*w*) (or *ρ*_k_) is expected to drop with increased burden due to density-dependent crowding effects. Following Anderson & Medley’s approach [29, 30], we have used the exponential decay function

2 $$ \rho (w)={\rho}_0{e}^{-w/{w}_0};\kern0.5em \mathrm{or}\kern0.5em {\rho}_k=\rho \left(k\kern0.5em \varDelta w\right)={\rho}_0{e}^{-k/{k}_0} $$

with maximal value *ρ*_0_, and threshold burden, *w*_0_, or ${k}_0=\frac{w_0}{\varDelta w}$ (for *h*_*k*_) to simulate this phenomenon. Equations (1) and (2) predict mean egg-release by each host carrying *w* worms

3 $$ E(w)=\rho (w)\phi (w);\kern0.5em \mathrm{o}\mathrm{r}\kern0.5em {E}_k={\rho}_k{\phi}_k\kern0.5em \mathrm{f}\mathrm{o}\mathrm{r}\kern0.5em {h}_k $$

**Fig. 2:**
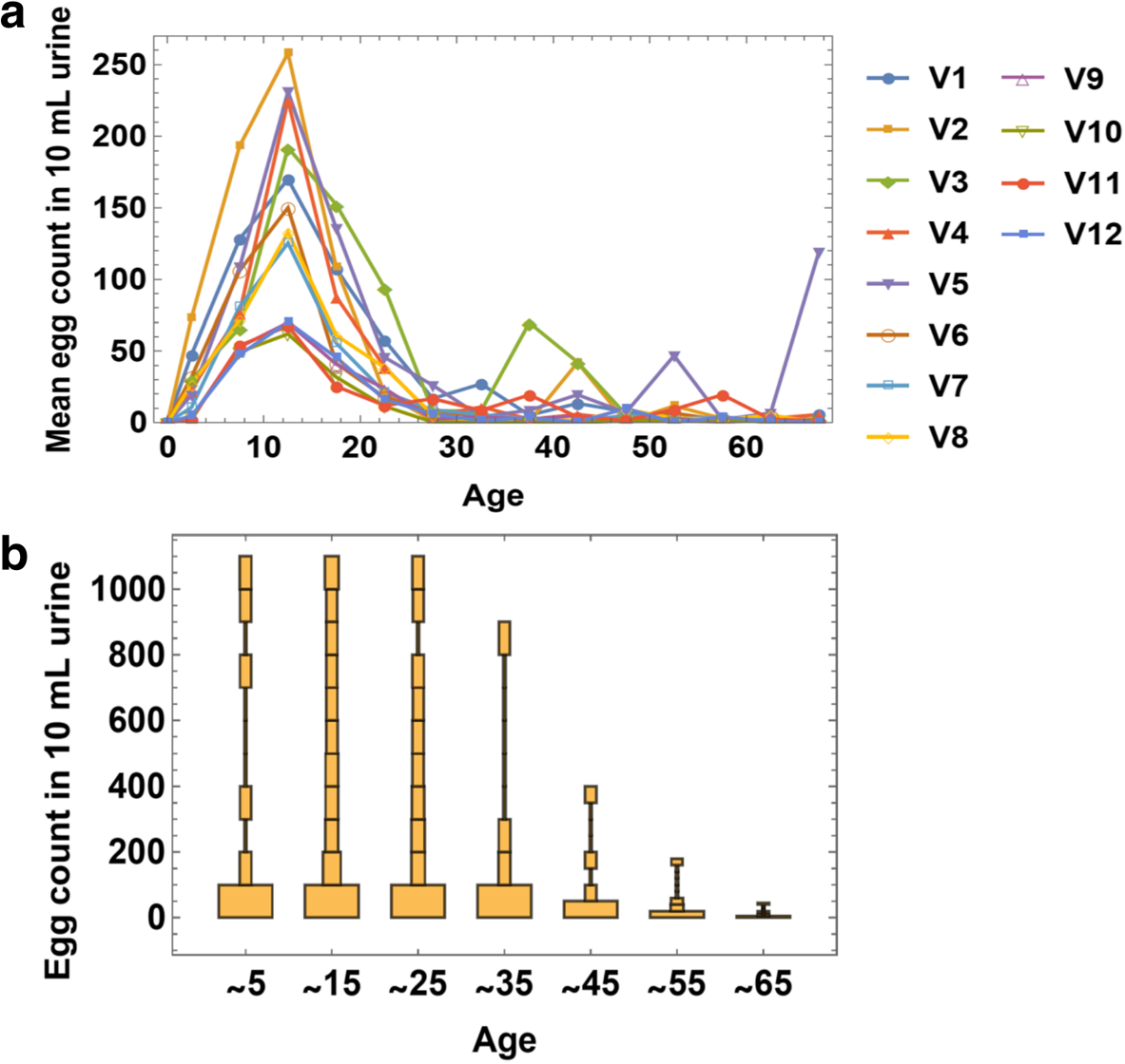
Uneven distribution of infection levels by village and by age groups. **a** Age distribution of mean intensity (egg count) for *S. haematobium* infection in 12 Kenyan villages, designated V1 to V12. **b** Egg-count distributions for different age groups exhibit overdispersed patterns. The orange rectangles stacked above each age range represent the relative prevalence of each infection intensity subgroup (binned by 100s of eggs per 10 ml urine) with subgroup prevalence reflected by the width of each rectangle

**Fig. 3:**
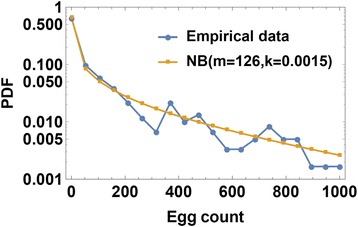
Typical community-wide egg-count distribution [24–27] fitted to a negative binomial distribution. The data from children (0–20 years of age) in the highest prevalence village, Milalani (V1), are plotted (*blue line and circles*) along with a fitted negative binomial (NB) curve (*yellow line and circles*) approximating the observed egg count distribution

Observed egg-counts, e.g. diagnostic test data, are typically over-dispersed (Fig. 2) and can be highly variable from day to day. A negative binomial (NB) distribution of daily egg output per worm has been proposed [16], and we adopt this premise at the level of individual egg output per host (Fig. 3). Specifically each fertilized female in *h*_*k*_-stratum is assumed to provide a random (NB) daily egg-count in the stool with mean *ρ*_*k*_ and aggregation *r.* Then egg-release by a given *h*_*k*_-host (carrying *ϕ*_*k*_ fertilized worms) is also negative binomial, with mean *E*_*k*_ = *ρ*_*k*_*ϕ*_*k*_ and aggregation *r*_*k*_ = *rϕ*_*k*_. For the overall SWB community with strata {*h*_*k*_}, egg-test results are random samples drawn from the mixed NB distribution (Table 3).

**Table 3:** Random egg-release

Egg release by mated females and individual hosts depends on worm fecundity *ρ*_*k*_, and mated-couple count *ϕ*_*k*_ (for *h*_*k*_ -stratum). The former is given by crowding function ${\rho}_k={\rho}_0\;{e}^{-k/{k}_0}$, with maximal value *ρ*_0_ and threshold *k*_0_. The latter *ϕ*_*k*_ can be estimated by assuming binomial sex-ratio distribution in the “*w*-strata” (*w* adult worms), Eq. (1). The predicted egg-release by *h*_*k*_-hosts, *E*_*k*_ = *ρ*_*k*_ *ϕ*_*k*_, gives its mean (expected) value used as measure of host infectivity. The actual release should be random (NB) with mean *E*_*k*_ and aggregation *r*_*k*_ = *r ϕ*_*k*_. Individual egg-counts-counts by all SWB hosts (strata {*h*_*k*_}) generate a mixed NB-distribution (4), illustrated in the schematic plot above. Each simulated egg-test of SWB community is then a random sample of size *H* (sampled pool) drawn from distribution *P*_*SW*_	

4 $$ {D}_M={\displaystyle {\sum}_{k=0}^n{h}_k\kern0.5em NB\left({E}_k\Big|r\kern0.5em {\phi}_k\right)} $$

In our SWB model development, simulated (random) community egg-tests were used extensively for model calibration and later for prediction and analysis of control intervention outcomes.

Unlike the results of individual diagnostic tests, which influence estimates of human infection prevalence, environmental egg-release by SWB community was considered a deterministic process that accumulates the random contributions of multiple hosts. Hence, in determining human-to-snail FOI, random egg-release (Eq. 4) is replaced by its mean value

5 $$ E={\displaystyle {\sum}_{k=1}^n{\rho}_k{\phi}_k{h}_k={\displaystyle {\sum}_{k=1}^n{E}_k{h}_k}} $$

and function *E* represents average host infectivity of SWB community {*h*_*k*_}.

### Details of the local snail population model

The snail dynamics modeling combines snail population biology and infection processes (Table 4). We assume the snail population obeys a logistic growth with maximal reproduction rate, *β*_*0*_, and carrying capacity, *K*, following models that are commonly used in population biology to account for growth in resource-limited environments. For modeling *Schistosoma* transmission within snail populations, three compartments (susceptible-exposed-infected (SEI)) are used, labeled as: *x*, susceptible; *y*, pre-patent infected; and *z*, patent/shedding infected, with total population *N* = *x* + *y* + *z*. It is assumed that shedding snails do not reproduce [31].

**Table 4:** Snail population-transmission dynamics

*Dynamic variables* for the snail model are population densities (per unit habitat) *x*: susceptible; *y*: prepatent; *z*: patent; *N = x + y + z*- total. $$ \overset{\beta }{\to}\underset{\begin{array}{l}\downarrow \\ {}\nu \end{array}}{x}\underset{\left(1-c\right)r}{\overset{\varLambda }{\rightleftarrows }}\kern0.24em \underset{\begin{array}{l}\downarrow \\ {}\nu \end{array}}{y}\overset{c\;r}{\to}\underset{\begin{array}{l}\downarrow \\ {}\nu \end{array}}{z} $$ *Basic processes* and parameters include (i) snail reproduction (logistic growth) *β* = *β*_0_(*x* + *y*)(1 − *N*/*K*), with maximal reproduction rate *β*_*0*_ and carrying capacity *K*; (ii) snail mortality *v*; (iii) snail FOI Λ(determined by human host egg outputs) ; (iv) recovery rate *r* (prepatency period 1/*r*) (v) patency conversion fraction *c*. In population growth term *β*, only susceptible and prepatent snails (*x* + *y*) reproduce. Combined growth-SEI dynamics consists of 3 differential equations $$ \begin{array}{l}\frac{dx}{dt}=\beta -\varLambda\;x-\nu\;x+r\left(1-c\right)y\\ {}\frac{dy}{dt}=\varLambda\;x-\left(r+\nu \right)\;y\\ {}\frac{dz}{dt}=c\;r\;y-\nu\;z\end{array} $$ Parameter values and ranges for the snail system are given in Table 5. Short-lived larval stages (*M, C*) equilibrate rapidly at levels proportion to human/snail (*H, N*) multiplied by their respective infectivity. Specifically, C* = *α*_*C*_ *N z*; M* = *β*_*M*_ *ω H E* (6) where ${\alpha}_C=\frac{\pi_C}{\nu_C}$ (“C-production /patent snail” over “C-mortality”). For miracidia the relevant inputs include environmental egg-release by host population *ω H E*, *ω* = human-snail contact rate, *H* - population size, *E* - mean host infectivity - egg release (Eq. 5), coefficient ${\beta}_M=\frac{\sigma_M}{\nu_M}$ (“survival fraction of eggs” over “M - mortality”)	

Here a successful miracidial invasion (Table 4) is followed by the prepatent stage lasting 1/*r* days, after which a fraction *c* of prepatent snails convert to the patent/shedding stage (z), while the remaining fraction (1 − *c*) may resolve infection to return to the susceptible (*x*) stage. Snail FOI Λ is determined by human infectivity (*E*), the human population size, and human-snail contact rate, ω.

### Human-to-snail force of infection in coupled SWB systems

Human-snail transmission is mediated by two larval stages, cercaria (*C)* and miracidium *(M*), which determine snail-to-human FOI (*λ*) and human-to snail FOI (Λ), respectively. It is convenient to measure all populations (human *H*, snail *N*, *M*, and *C*) by their densities per unit habitat.

Human and snail FOI are determined by larval equilibria (Eq. (6) of Table 4), but their functional forms require more detailed analysis. For human FOI (rate of worm accumulation) we expect a linear dependence on *C*, proportional to patent snail prevalence. Hence

7 $$ \lambda =\alpha \omega \kern0.5em N\;z $$

for exposure rate *ω*, patent snail density *N z*, and (snail-to-human) transmission coefficient *α* that accounts for intermediate cercaria stage, and the probability of worm establishment in a human host. For snail FOI, many conventional modeling approaches have adopted a similar linear relation *Λ* = *bωH E* with (human-to-snail) transmission coefficient *b*, but this relation is questionable. Snail infection is accounted by prevalence variables (*y, z*), and the functional relation between miracidial density (*M*) and FOI Λ requires a more careful analysis. We propose, instead, that the link is a nonlinear function that takes into account two processes: (i) multiple possible *M*-invasions [32] and (ii) sporocyst establishment in susceptible snails [32]. The invasion process likely depends on the average number of miracidia per snail, *M*/*N*, and a snail innate resistance level, *p* = the probability of ejecting an invading miracidium. Multiple biological and environmental factors could contribute to a successful miracidial invasion, and *p* serves as a crude proxy for their cumulative (mean) effect in the susceptible snail population. Having fixed *M/N*, we estimate the fraction of successfully invaded snails by 1 − *ρ*^*M*/*N*^. The resulting snail FOI is the product of *Λ*_0_ - the rate of sporocyst development in snails (minimum 1–2 weeks [31, 32]), and the invaded snail fraction:

8 $$ \varLambda ={\varLambda}_{\mathsf{0}}\left(\mathsf{1}-{p}^{M/N}\right)\kern0.5em ; $$

It is convenient to replace snail resistance *p* = *e*^− *α*^ by susceptibility parameter, *α* = 1n(1/*p*), and using miracidia equilibrium M* = *β*_*M*_*ωH E* (Eq. 6) to write Λ as function of human infectivity *E.*

9 $$ \varLambda ={\varLambda}_{\mathsf{0}}\left(\mathsf{1}- \exp \left[\hbox{-} b\omega \frac{H\;E}{N}\right]\right) $$

The key inputs in Λ are sporocyst establishment rate Λ_0_ and the transmission coefficient *b* = *αβ*_*M*_ (product of “miracidium coefficient” *β*_*M*_ times “snail susceptibility” *α*). At low transmission intensity (*H E* ≪ 1) function (Eq. 9) is approximately linear $\left(\varLambda \approx {\varLambda}_0b\omega \frac{H\;E}{N}\right)$ - the conventional form of snail FOI. But unlike a “linear” Λ function, Eq. (9) would saturate at a maximum level = Λ_0_ for large *H E*. This has important implications for transmission dynamics and model calibration. In general, one could expect higher values of estimated coefficient *b*, compared to the linear model case. In dynamic simulations, this would yield higher persistence of transmission and a more rapid rate of human reinfection after mass drug administration (MDA) to the human population.

Thus, the basic inputs needed for running a coupled SWB model system are: (i) the biological SWB parameters of worm fecundity *ρ*(*w*) and egg-release *E*_;_ (ii) human and snail population densities *H, N*, along with their demographic (birth, death, migration) parameters; and (iii) the human exposure/ water contact rate *ω*, and the resulting operative transmission parameters (*α, b*).

### Calibrating the human SWB system

The first input for calibration is a human egg-test data set (~500 cases) from a given community, or a population subgroup (e.g. age group). The goal of this human-side SWB calibration is to find most likely values of biological (fecundity) parameters *ρ*_*B*_ = {*ρ*_0_, *w*_0_, *r*} and transmission λ (= “rate of worm accumulation”/”worm mortality”) that are consistent with test data. Parameter λ is proportional to the mean worm burden of the SWB community in its equilibrium (endemic) state, $\overline{w}=\lambda \varDelta w$.

For each choice (*λ*, *ρ*_*B*_), we simulate an ensemble of random egg-tests outputs to compare with the observed data. Each simulated egg-test involves two random steps: (1) the random selection of subjects from a population sub-group to be tested and (2) the random egg-release by each tested host in each stratum. These two steps are combined via a random sampling of a mixed NB distribution (Eq. 4) with prescribed parameters {*λ*, *ρ*_*B*_ = (*ρ*_0_, *k*_0_, *r*)} (see Table 3). By this approach, we generate an SWB equilibrium {*h*_*k*_(*λ*)} from *λ* and then compute the mean egg-release *E*_*k*_ = *ρ*_*k*_*ϕ*_*k*_ and aggregation *r*_*k*_ = *rϕ*_*k*_ for each stratum *h*_*k*_, using the determined biological parameters *ρ*_*B*_, i.e. ${\rho}_k={\rho}_0{e}^{-k/{k}_0}$ (fecundity) and *Φ*_*k*_ (mating (Eq. 1)). Three inputs {*h*_*k*_, *E*_*k*_, *r*_*k*_} give rise to the mixed NB-distribution *D*_*M*_ (*λ*, *ρ*_*B*_) of (Eq. 4). The simulated community egg-test output is thus a random sample of *H* subjects (the surveyed pool) drawn from distribution ${D}_M,{\mathsf{E}}_T=\left\{{e}_1;\dots; {e}_H\right\}$ (*e*_*i*_ - egg-count of *i*-th test sample).

For further parameter fitting (calibration), individual counts are binned into egg-count distributions *E*_*S*_ = {*c*_0_, *c*_1_, …} determined by prescribed sequence of *E*-partition bins: *E*_0_ = 0 < *E*_1_ = 1 < *E*_2_ < … < *E*_*n*_
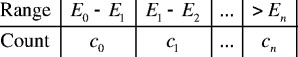


Here, *c*_0_ counts negatives (uninfected pool), *c*_1_ - includes the range *E*_1_ ≤ *e* < *E*_2_, etc. Different binning choices *E*_0_ < *E*_1_ < *E*_2_ < … are possible depending on the range and distribution of test diagnostics. For *S. haematobium*, 10 ml urine filtration [21] has typical counts 0 ≤ *e* ≤ 1000, and egg count values are highly over-dispersed (Fig. 3). A suitable choice in such context is the log-scale *E*_*k*_ = 2^*k*^(*k* = 1, 2, …, 10), such that the egg-count range, ([0–1,000), would split into 10 log-scale bins.

**Fig. 4:**
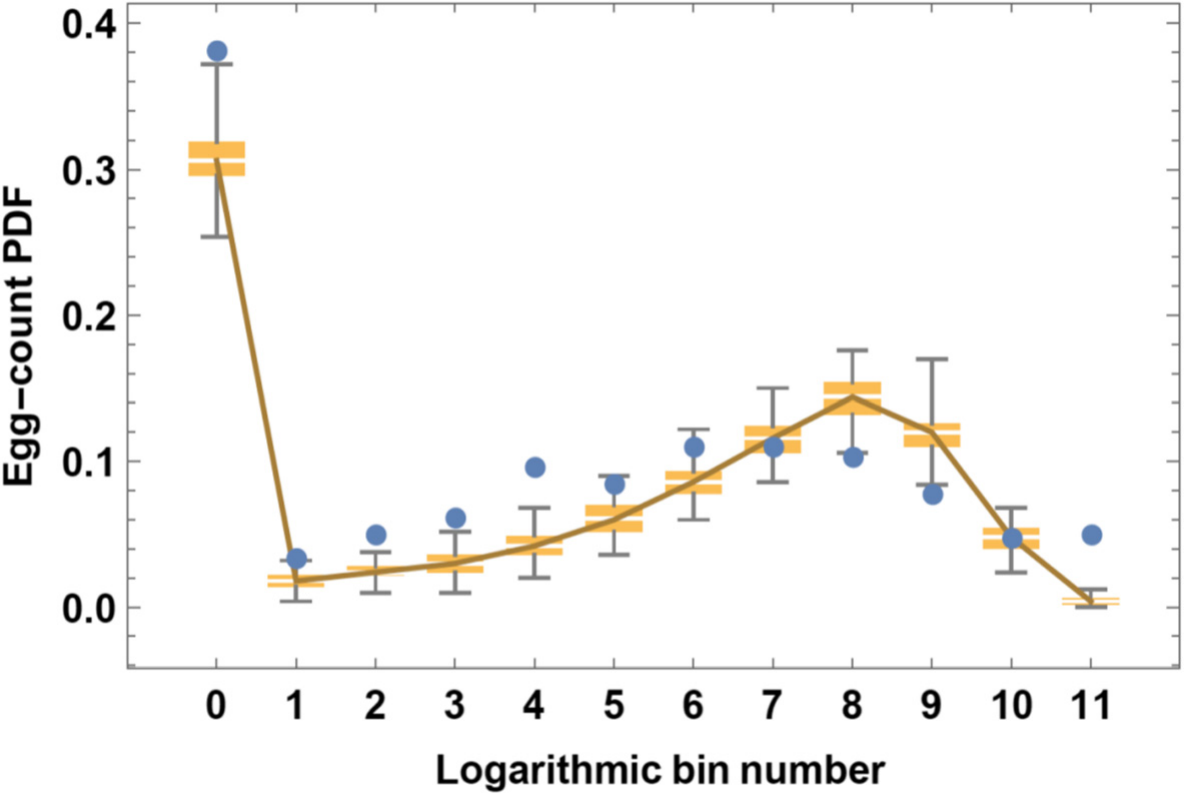
Simulated egg-test ensemble distributions *vs* egg-count data for a high risk village. A logarithmic bin scale (*E*_*k*_ = 2^*k*^, *k* = 0, 1, …, 11) was used to plot aggregated patient data (*blue dots*) for comparison to results from multiple, data-generating, random SWB test simulations. Here a simulated egg-test ensemble (200 random realizations) was created based on a fixed choice of model parameters (*λ* = 1.8, *ρ*_0_ = 27, *w*_0_ = 100, *r* = .11). Simulation results are represented by a box and whisker plot that shows median and 25–75 % quartiles, and the 95 % range of the simulations, plotted by egg-count bin number (logarithmic scale)

The log-bin counts of test data and of simulated tests show a typical bimodal pattern, with maximal value *c*_0_ (uninfected) and another peak between 50 < *E* < 500 (Fig. 4), depending on mean community burden. The elevated negative egg count category, *c*_0_, could be an overestimate due to low test sensitivity for light infections [20, 21], or it could mean low infection prevalence among the tested subject pool.

Simulated egg distribution *E*_*S*_(*λ*, *ρ*_*B*_) (Eq. 10) depends on model parameters, but each output *E*_*S*_ = {*c*_0_, *c*_1_, …} is random. The same, we expect, should hold for the real test data *E*_*D*_ = {*d*_*k*_}, due to uncertainties of sampling and diagnostics [17–22]. Our goal is to compare two random samples (*E*_*S*_; *E*_*D*_) to assess their “proximity” via a suitable distance-function, *d*(*E*_*S*_; *E*_*D*_). The task is confounded by randomness of both samples, particularly of simulated *E*_*S*_. To account for this, we assess the “proximity” between the simulated test ensemble $\mathsf{E}=\mathsf{E}\left(\uplambda, {\uprho}_{\mathrm{B}}\right)=\left\{{\mathrm{E}}_{\mathrm{S}}\right\}$, and observed data, *E*_*D*_, instead of comparing individual test samples {*E*_*S*_}. Such a procedure is cast in a Bayesian framework by asking how likely it is to observe a given data set *E*_*D*_ for a particular (parameter choice) ensemble $\left(\mathsf{E}=\mathsf{E}\left(\lambda, {\rho}_B\right)\right)$. In other words, we ask what is the probability of observing *E*_*D*_ conditioned on $\mathsf{E}?$.

A natural answer can be given via the mean and covariance structure of ensemble $\mathsf{E}\left(\lambda, {\rho}_B\right)$, {*Ē*(*λ*, *ρ*_*B*_), *σ*(*λ*, *ρ*_*B*_)}. Specifically, we define the distance (error) function between $\mathsf{E}$ and *E*_*D*_ as:

11 $$ d\left(\mathsf{E},\ {E}_D\right)=\left(\overline{E}-{E}_D\right)\;{\sigma}^{-1}\;\left(\overline{E}-{E}_D\right). $$

Definition (Eq. 11) gives a family of *likelihood* weights on parameter space (λ, *ρ*_*B*_)

12 $$ W=W\left(\lambda, {\rho}_B\right)={e}^{-d\left(\mathit{\mathsf{E}},{E}_D\right)} $$

with larger *W* corresponding to more likely parameter choices, consistent with the data *E*_*D*_ (see Additional file 3 for technical details).

Calibration proceeds by randomly scanning the parameter space (*λ*, *ρ*_*B*_) (“uninformed” prior) and generating a mean-covariance structure for uniformly sampled 4D hypercube in the parameter space: *λ* ' < *λ* < *λ* "; *ρ*_0_ ' < *ρ*_0_ < *ρ*_0_ "; *k*_0_ ' < *k*_0_ < *k*_0_ "; *r* ' < *r* < *r* ".

Once such a mean-covariance test-bed ({*Ē*(*λ*, *ρ*_*B*_), *σ*(*λ*, *ρ*_*B*_)}) is computed, any specific test data (*E*_*D*_) will generate a family of likelihood weights (Eq. 12) on the (*λ*, *ρ*_*B*_) -space and give the empirical posterior distribution $\mathsf{D}=\mathsf{D}\left(\lambda, {\rho}_B\right)$ consistent with *E*_*D*_.

Figure 4 illustrates the range of likely egg output simulation runs using a fixed, random parameter choice that makes the *Ē*_*S*_, *E*_*D*_ distribution fairly close.

The above calibration procedure is applied to the youngest age group (children). The adult calibration requires an additional input, namely a SWB - source term coming from maturing children (see Additional file 2). Thus, the adult parameter space has an additional calibrated parameter - pre-adult *λ*_*C*_, along with adult *λ*_*A*_ and biological adult *ρ*_*B*_ = (*ρ*_0_, *k*_0_, *r*). To estimate its likelihood weights, we proceed as above by scanning the extended parameter space (*λ*_*C*_, *λ*_*A*_, *ρ*_*B*_) and generating the adult test-bed {*Ē*(*λ*_*C*_, *λ*_*A*_, *ρ*_*B*_), *σ*(*λ*_*C*_, *λ*_*A*_, *ρ*_*B*_)}.

Once the mean-covariance test-bed is computed, calibration of any specific data set proceeds straightforwardly. Binned egg-data is substituted into the error function (Eq. 11) to get a family of likelihood weights (Eq. 12), resulting in an (empirical) posterior distribution consistent with the data.

Given a community (village) test data, we can generate two (child and adult) posterior distributions on their respective parameter spaces in 2 steps. First, the child group is calibrated to get its posterior and the resulting marginal distribution of child FOI (weights {*W* (*λ*_*C*_)}). Then the adult posterior is computed using source terms derived from the child SWB strata {h^*^(*λ*_*C*_)} evaluated at properly weighted FOI *λ*_*C*_, i.e. the distribution *W* (*λ*_*C*_) estimated earlier. The resulting adult likelihood weights in the extended parameter space are conditioned on the likely child values *W* (*λ*_*C*_), as

$$ W\left({\lambda}_C,{\lambda}_A,{\rho}_B\right)=W\left({\lambda}_C\right)\cdot {e}^{-d\left(\mathit{\mathsf{E}},{E}_D\right)} $$

### Calibrating the coupled human-snail system’s transmission coefficients

Having calibrated the human side of SWB dynamics, our next task is to combine human and snail infection data to estimate transmission coefficients (*α*, *b*) of coupled human-snail systems. In earlier work [14], we developed such a scheme for simplified SWB - snail systems. The current SWB modelling approach required significant modification, as outlined below.

First we demonstrate calibration for a single human SWB - single snail site, then proceed to more complex, coupled child-adult systems. Equilibrium solutions of the snail system (Additional file 4) allow us to relate observed snail infection data to model parameters. Typical data include snail prevalences (prepatent - *y*, patent - *z*, total - *N*) expressed through basic inputs: growth rate *β*_*0*_, mortality $\nu$, carrying capacity *K*, as well as snail FOI Λ (which needs to be estimated during calibration). Some unknown (uncertain) parameters, such as the local snail carrying capacity *K* (assumed here to be stationary), the human-snail contact rate *ω*, and snail susceptibility *α*, can be combined into single transmission coefficient. Specifically,

i) $A=\alpha \omega =\frac{\lambda /\varDelta w}{N\;z}$ - snail-to-human transmission
ii) $c=\frac{\nu }{r}\frac{z}{y}$ - patency conversion fraction (estimated from snail prevalences)
iii) $\varLambda =\frac{v\left(v+r\right){y}^{*}}{v-\left(c\;r+v\right){y}^{*}}$ - snail FOI, estimated from snail prevalence data, known *ν, r* and estimated *c*
iv) $B=b\omega =\frac{N^{*}\left(\varLambda \right)}{N\;E}1\mathrm{n}\left(\frac{\varLambda_0}{\varLambda_0-\varLambda}\right)$ - human-to-snail transmission in snail FOI (Eq. 9).

Here N^*^ (Λ) is total equilibrium snail density (see Additional file 4), and Λ_0_ is rate of sporocyst establishment in snails (Table 5).

**Table 5:** Demographic and biological parameters for SWB systems

Parameters	Name	Value
Host turnover rates:		
Child	*μ*_*C*_ = *τ* + *δ*_*C*_ (maturation + mortality)	0.05 + 0.003/year
Adult	*μ*_*A*_ (mortality)	0.02 – 0.03/year
Demographic sources:		
Child	*S* ^*C*^ = {*b*_*c*_, 0, 0, …}; per capita birth rate	*b*_*C*_ = 0.032/year [37]
Adult	*S* ^*A*^ = *τ*{*h*_0_^*C*^, *h*_1_^*C*^, …}	
Mean daily urine release [34]:		
Child		*U*_*C*_ = 1100 ml
Adult		*U*_*A*_ = 1300 ml
Worm turnover rate:		
Worm mortality	*γ*	0.2/year [50]
Snail parameters:		
Snail mortality	*v*_*S*_	2.6/ year
Recovery/conversion rate	*r*	$\frac{1}{4}$ weeks
Patency conversion fraction	*c*	0.05–0.20
Rate of sporocyst establishment	Λ_0_	$\frac{1}{2}$ weeks

We first estimate (*c*, Λ) - equations (ii-iii), then apply them to transmission coefficients *A, B* via equations (i-iv). Calibration of a mixed SWB-system, made of several groups (*i* = 1, 2, …), requires additional assumptions on relative transmission rates *B*_*i*_*/A*_*i*_. Namely, *B*_*i*_/*A*_*i*_ = *b*/*α* should be identical for all population groups *i* = 1, 2, …. To compute *b*/*α* we use estimated transmission coefficients ${A}_i=\frac{\lambda_i}{N\;z}$ and replace human infectivity factor *H E* in equation (iv) by combined infectivity of all population groups ∑*H*_*i*_*E*_*i*_. Then

$$ \frac{b}{\alpha }=\frac{N^{*}\left(\varLambda \right)}{{{\displaystyle {\sum}_i{\alpha}_iH}}_i{E}_i}1\mathrm{n}\left(\frac{\varLambda_0}{\varLambda_0-\varLambda}\right) $$

and human-to snail transmission by each group is given by ${B}_i=\frac{b}{\alpha }{A}_i$.

### Adding MDA-based control to the SWB system

The effect of drug treatment on stratified (SWB) population is to move a treated fraction of stratum *h*_*n*_ (*t*) to a lower-level stratum *h*_*m*_ (*t*), where *m ≈ ε n* is determined by the estimated efficacy of drug *ε* = fraction of adult worms surviving each drug treatment (see [14, 33]). In particular, all strata in the lowest range {*h*_*m*_ : 0 ≤ *m* < 1/*ε*} shift to *h*_*0*_ (effective clearing), the next interval {*h*_*m*_ : 1/*ε* ≤ *m* < 2/*ε*} would go to *h*_1_, etc. In numeric code, each drug treatment is simulated as an instantaneous event, due to the short duration of drug action (days) compared to the slow time-scale of transmission dynamics (months to years). Computationally, terminal values of SWB variables at the treatment time *t*_0_ are reinitialized to new (post-treatment) values, depending on MDA inputs, the treatment coverage fraction (0 < *f* < 1), the drug efficacy, *ε*, etc. Each MDA- “event” would then reshuffle variables {*h*_*m*_(*t*)} according to

$$ \begin{array}{l}{h}_0\Big|{}_{t_0}=\left(1-f\right){h}_0+f{\displaystyle \sum_{0\le m<1/\varepsilon }{h}_m\left({t}_0\right)}\hfill \\ {}{h}_1\Big|{}_{t_0}=\left(1-f\right){h}_1+f{\displaystyle \sum_{1/\varepsilon \le m<2/\varepsilon }{h}_m\left({t}_0\right)}\hfill \\ {}{h}_2\Big|{}_{t_0}=\left(1-f\right){h}_2+f{\displaystyle \sum_{2/\varepsilon \le m<3/\varepsilon }{h}_m\left({t}_0\right)}\hfill \\ {}\dots \hfill \end{array} $$

The reinitialized system is solved over the prescribed time-range (between two “events”) and the process continues.For more detail, see Fig. 4 of reference [33].

## Results

### Model calibration

We applied our calibration scheme to infection data collected in the Msambweni region of Coastal Kenya [23–26], where repeated cross-sectional surveys were conducted in 12 villages using standard filtration diagnostics (10 ml urine sample test [20, 21]) along with surveys of water contact [26] and local snail infection data [24]. The first rows of Table 6 (children) and Table 7 (adults) summarize basic demographics and epidemiological results of those surveys. For convenience, we ordered villages by their infection prevalence from highest risk (*V*_1_) to lowest risk (*V*_12_) based on initially observed childhood prevalence values.

**Table 6:** Calibration results for the children’s age group: demographic and infection data with calibrated model parameters (mean ± SD) for 12 Msambweni villages

Villages	V1	V2	V3	V4	V5	V6	V7	V8	V9	V10	V11	V12
Population	602	286	189	281	353	638	557	921	944	720	815	803
Prevalence (%)	71	67	52	51	50	49	43	32	27	25	24	23
Mean Intensity	126	170	113	116	126	91	77	78	46	48	49	46
Human FOI^a^	5.9 ± 1.3	5.6 ± 1.4	4.5 ± 1.6	3.2 ± 1.2	4.1 ± 1.5	4.2 ± 1.6	2.3 ± 0.9	2.1 ± 1.3	1.8 ± 1.1	1.9 ± 1.3	2.3 ± 1.5	1.4 ± 1.0
Maximum fecundity variable, ρ_0_	25 ± 8	43 ± 11	36 ± 13	44 ± 13	45 ± 13	33 ± 13	36 ± 12	48 ± 15	40 ± 15	45 ± 15	45 ± 15	46 ± 14
Crowding threshold variable, w_0_	116 ± 47	127 ± 42	118 ± 43	123 ± 43	123 ± 42	118 ± 43	123 ± 43	128 ± 43	125 ± 45	130 ± 45	130 ± 44	129 ± 46
Aggregation	0.03 ± 0.01	0.04 ± 0.02	0.03 ± 0.02	0.05 ± 0.02	0.03 ± 0.02	0.03 ± 0.02	0.06 ± 0.02	0.05 ± 0.03	0.05 ± 0.03	0.04 ± 0.02	0.03 ± 0.02	0.05 ± 0.03

^a^The mean rate of worm accumulation per year

**Table 7:** Calibration results for adults: demographic and infection data with calibrated model parameters (mean ± SD) for 12 Msambweni villages

Villages	V1	V2	V3	V4	V5	V6	V7	V8	V9	V10	V11	V12
Population	508	264	189	247	259	530	472	653	738	456	512	746
Prevalence (%)	33	24	31	23	27	19	14	13	14	7	13	12
Mean Intensity	19	11	30	11	25	6	8	10	7	2	10	6
Human FOI^a^	1.6 ± 0.6	1.2 ± 0.6	1.6 ± 0.6	1.2 ± 0.6	1.4 ± 0.6	1.0 ± 0.6	0.9 ± 0.7	0.7 ± 0.6	0.8 ± 0.6	0.5 ± 0.6	0.7 ± 0.7	0.7 ± 0.6
Maximum fecundity variable, ρ_0_	11 ± 5	4 ± 3	11 ± 6	6 ± 4	10 ± 6	5 ± 4	11 ± 6	12 ± 6	8 ± 6	11 ± 7	13 ± 6	11 ± 6
Crowding threshold variable, w_0_	122 ± 44	122 ± 43	125 ± 43	125 ± 44	124 ± 43	123 ± 44	120 ± 44	120 ± 44	119 ± 44	126 ± 44	122 ± 44	120 ± 44
Aggregation	0.03 ± 0.02	0.05 ± 0.03	0.04 ± 0.02	0.04 ± 0.02	0.04 ± 0.02	0.04 ± 0.03	0.03 ± 0.02	0.04 ± 0.03	0.04 ± 0.03	0.03 ± 0.02	0.03 ± 0.02	0.04 ± 0.02
Pre-adult FOI	3.7 ± 2.3	3.7 ± 2.3	4.0 ± 2.4	3.7 ± 2.3	3.8 ± 2.4	3.7 ± 2.2	3.7 ± 2.3	3.8 ± 2.3	3.8 ± 2.3	3.3 ± 2.2	3.9 ± 2.3	3.8 ± 2.3

^a^The mean rate of worm accumulation per year

For data analysis and model calibration, the total population of each village was split into children (0–20 years) and adults (20+); this choice was partly motivated by distinctive drop of infection about age 20 (Fig. 2). Additional inputs specific for Kenya are listed in Table 5. Two sets of calibrated parameters (Table 8) include age-specific fecundity (*ρ*, *w*_0_, *r*) and human FOI *λ*. The final result of calibration was 24 posterior distributions, i.e. the 12 Msambweni villages, with two modeled age groups for each village (Tables 6 and 7).

**Table 8:** Parameters and their expected ranges based on calibration

Parameter	Symbol [units]	Value range
Force of infection (FOI)	*λ* [worm/year]	Child: (0, 8)
		Adult: (0, 3)
Maximum egg release	*ρ*_0_ [egg/female]	Child: (10, 70)
		Adult: (1, 25)
Crowding threshold	*w*_0_ [worm]	(50, 200)
Egg aggregation^a,b^	*r* ^a^	(0, 0.1)
Child FOI for adult calibration	*λ*_*C*_ [worm/year]	(0, 8)

^a^Dimensionless parameter

^b^Aggregation factor for daily egg counts when modelled as a negative binomial distribution

Posterior distributions and their likelihood weights (Eq. 12) play important roles in our analysis and the control simulations reported below. All statistical outputs (means, correlations, quantiles, etc.) were computed relative to ensemble $\mathsf{D}$. Thus for MDA simulations, we ran multiple treatment histories using a suite of likely parameter choices (for a given community) based on $\mathsf{D}$, and assigned each output its respective likelihood weight.

**Fig. 5:**
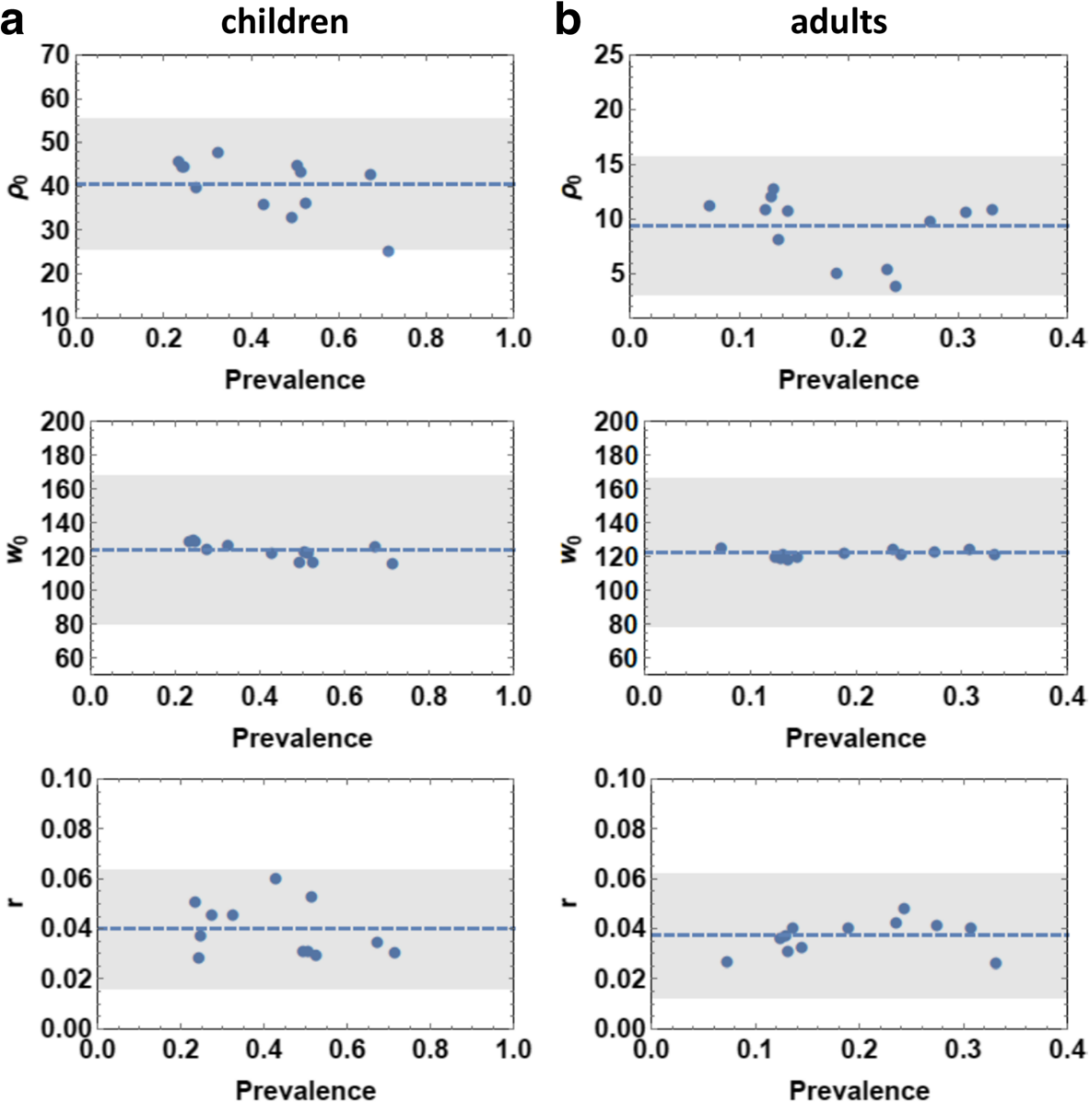
Estimated ensemble mean biological parameters for twelve Msambweni villages, plotted against observed egg-count prevalence data. The left column (**a**) has maximal egg release (*ρ*_0_), crowding (*w*_0_), and aggregation (*r*) mean parameter values for children (0 to 20 years old) graphed together for every village; the right column (**b**) has values for adults. Dashed lines are linear regressions for the twelve village values for each parameter

**Fig. 6:**
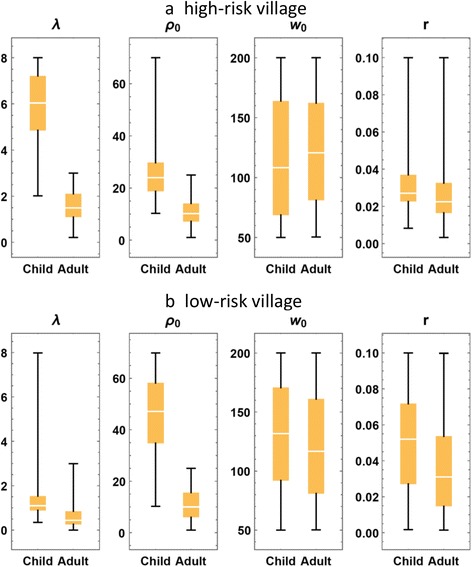
Box plot comparison of the median and range of calibrated model parameters. Summary ranges of estimated values for maximal egg release (*ρ*_0_), crowding (*w*_0_), and aggregation (*r*) parameters, presented for children and adults in high transmission villages (**a** upper panel) and low transmission villages (**b** lower panel)

A brief summary of calibration results for the 12 villages (ensemble mean and standard deviation, SD) is given in Table 6 (for children), Table 7 (for adults), and the accompanying Fig. 5 (showing ensemble-mean biological parameters). Figure 6 shows the distributions of calibrated parameter for child and adult groups in high and low transmission villages.

Not unexpectedly, fecundity parameters exhibited fairly consistent values across the region with nearly horizontal linear fits for these parameter values across villages (Fig. 5). This suggested that hosts carrying comparable worm burden release similar egg-counts regardless of residing in high vs. low transmission areas. The only significant difference in parameter estimates comes between age groups, with children showing much higher, per-worm fecundity than adults (*ρ*_*C*_ ≫ *ρ*_*A*_). Consistent values of biological parameters across the region allow us to combine the 12 local village distributions of (*ρ*_0_, *w*_0_, *r*) into a single posterior ensemble, ${\mathsf{D}}_B$. This “biological ensemble” ${\mathsf{D}}_B$ is then used in the subsequent analysis and simulation of coupled human-snail systems. Calibrated child and adult FOIs (λ_C_ ; λ_A_) show consistently higher values for children than for adults across the region (see Fig. 6).

### Estimates for the density-dependent crowding effect

**Fig. 7:**
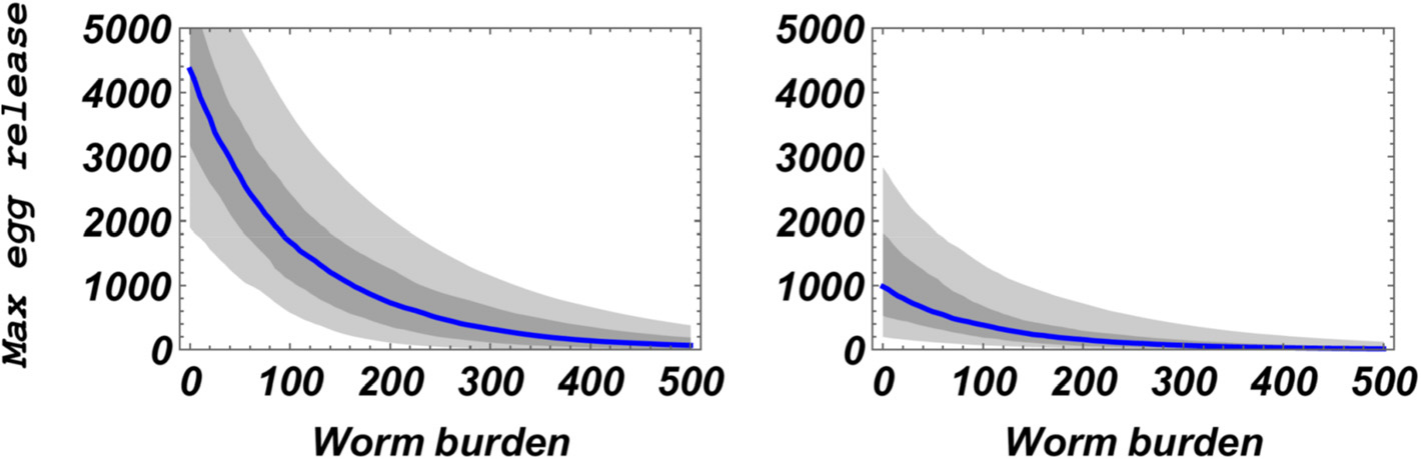
Density-dependent worm fecundity estimates [eggs/worm/day] for children (left panel) and adult (right panel) groups. Ensemble gray-scale envelopes shown here include median (*blue*), min/max (*light grey*), and 25-75 % quartile (*dark grey*) estimates of worm fecundity at different levels of individual human worm burden. 1,000 random parameter choices from SWB biological posterior ${\mathit{\mathsf{D}}}_B$ were used to generate the estimated crowding-effect curves

Our calibration results also provide estimates of the worm fecundity ‘crowding’ function *ρ* (*w*) of Eq. (2). The data on worm fecundity in human hosts are sparse and difficult to measure in vivo; however, our results give indirect estimates of *ρ*_*B*_ = (*ρ*_0_, *w*_0_, *r*) as part of biological ensemble ${\mathsf{D}}_B$. Ensemble envelopes of fecundity function (based on 1,000 random choices from biological posterior ${\mathsf{D}}_B$) are shown in Fig. 7. For these estimates, diagnostic test results (eggs per 10 ml urine) were adjusted for average daily urine release: *U*_*C*_ ≈ 700 ml (for children) and *U*_*A*_ ≈ 1300 (for adults) [34]. The estimated crowding effect was pronounced for both human age groups, with children’s subgroup worm-fecundity dropping from a high of > 1,000 eggs/worm/day at very low burden (in children) to fewer than 100 eggs/worm/day at heavy burden. For adults, the estimates were 1,200 eggs/worm/day at low intensity, declining with half-value every ≈ 120 worms of burden. Overall patterns were consistent with known data (see, e.g. reference [9], chapter 15, and [29]). An alternative approach to modeling fecundity effects was included in our earlier work using a simpler SWB without in-host biology [14]. There, fecundity was assumed uniform across all strata and the resulting calibrated *ρ*-values were broadly distributed in the range [0, *ρ*_0_]. The two models, simple SWB and the current refined version, differ in their predictions as described below.

### Predicting prevalence and intensity curves

The SWB model predicts specific relations between prevalence and intensity based on its simulated egg-test results. Namely, for each parameter choice (*λ*, *ρ*_0_, *k*_0_, *r*), we can take the corresponding SWB equilibrium {*h*_*k*_ (*λ*)} and compute its mating/fecundity factors *ρ*_*k*_, *ϕ*_*k*_ (Eqs.1 and 2) in terms of biological parameters *ρ*_*B*_ = (*ρ*_0_, *k*_0_, *r*). Then, from mixed-NB assumption (Eq. 4) on egg-release, we get formulae for prevalence *P*_*E*_ and infection intensity *M*_*E*_ as functions of parameters (*λ, ρ*_*B*_):

13 $$ \begin{array}{l}{P}_E\left(\lambda, {\rho}_B\right)=1-{\displaystyle \sum_{k=0}^n{\left(\frac{r}{r+{\rho}_k}\right)}^{r{\phi}_k}{h}_k\left(\lambda \right)}\hfill \\ {}{M}_E\left(\lambda, {\rho}_B\right)={\displaystyle \sum_{k=1}^n{\rho}_k{\phi}_k{h}_k\left(\lambda \right)}\hfill \end{array} $$

**Fig. 8:**
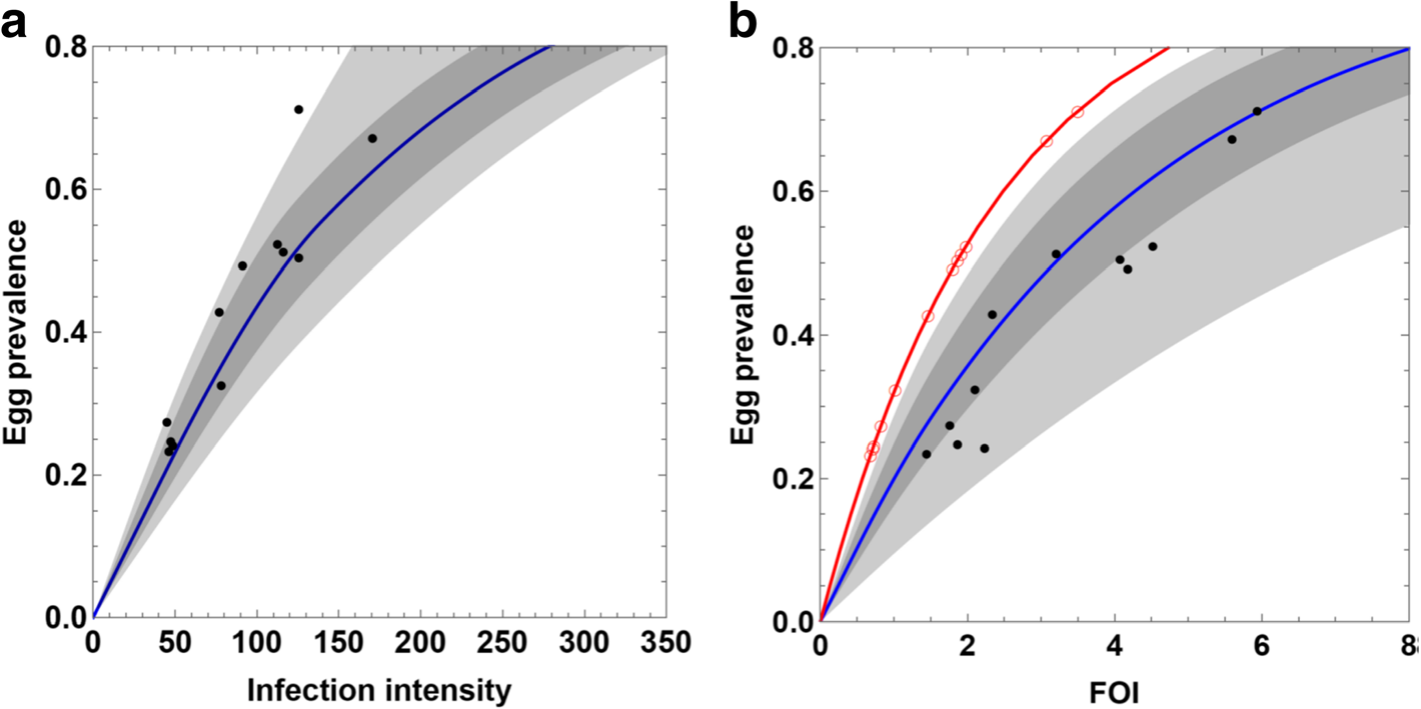
Prevalence-intensity and prevalence-FOI curves for observed data compared to SWB ensemble estimates. In panel **a**, intensity-prevalence curves (*P*_*E*_(*λ*), *M*_*E*_(*λ*)) over a range of *λ* values for posterior biological ensemble {*ρ*_*B*_} are plotted against the twelve Msambweni village data points: SWB ensemble median values (*blue curve*), their 25–75 % quantiles (*dark gray*), and 5–95 % quantiles (*light gray*) are shown. In panel **b**, FOI-prevalence curves are shown for *λ*(*P*_*E*_) (ensemble envelope) along with derived Msambweni data points; these dots show the estimated “ensemble mean” for individual village λ-values *vs* observed prevalence data for these 12 communities. The *red* curve shows the associated estimates for function λ, using the older, simpler SWB without correction for host-worm biological factors. An important implication of this discrepancy is that estimates of human FOI and transmission coefficients will be significantly underestimated if the model does not account for in-host biology

Each biological choice *P*_*B*_ gives a particular (parametric) prevalence-intensity curve (Eq.13), by continuously varying *λ*. Whereas equations (Eq. 13) define an increasing function *M*_*E*_ = *f*(*P*_*E*_), there is no simple analytic formula for it. Such curves can be computed and manipulated numerically, however. Fig. 8a compares the envelope of theoretical curves (Eq. 13) based on the biological posterior ensemble calibrated with Kenyan survey data [26]. Most data points for children lie within the 95 % quantile envelope, though we observe some departure (over-prediction) at higher prevalence values.

### Model validation

**Fig. 9:**
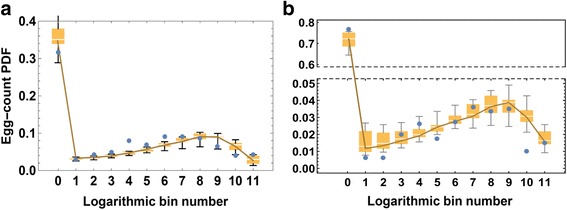
Validation of SWB model calibration for child and adult groups in a high transmission community. For each group (panel **a**, children; panel **b**, adults), we used its calibrated biological parameter ensemble and associated FOI *λ* estimates to generate a likely range of community realizations based on 200 different parameter choices, then simulating a likely egg-test distribution for each choice. These were then binned to indicate probable egg-count distributions. The bar-whisker chart of these binned counts is compared to the observed data (blue dots) for each group. The y-axis in panel **b** is truncated, having two different sections with two different scales

First, we generated an ensemble of virtual communities drawn from two calibrated posteriors. Then each group was randomly tested and its binned egg-counts recorded. The resulting ensemble of bin-counts were compared to the test data of each group, (see Fig. 9 for children and adult subgroups), which produced reasonable agreement with the data overall. Model prevalence estimates were close to the observed data points (i.e. within the 95 % uncertainty envelopes) for both child and adult groups.

Another validation test involved a functional relation between egg-test prevalence *P*_*E*_, FOI *λ* or its inverse function *λ*(*P*_*E*_), and predicted mean infection intensity (= mean egg-count). Model equations (Eq. 13) gives both *P*_*E*_(*λ*, *ρ*_*B*_) and *M*_*E*_(*λ*, *ρ*_*B*_) as functions of *λ* and biological parameters *ρ*_*B*_ = (*ρ*_0_, *w*_0_, *r*). To test consistency of such relations with village data across the region, we took a random selection of parameters {*ρ*_*B*_} drawn from the biological posterior ${\mathsf{D}}_B$ and for each choice computed *M*_*E*_(*λ*, *ρ*_*B*_) and *P*_*E*_(*λ*, *ρ*_*B*_) -curves and their envelopes, shown in Fig. 8a, b. We found the data points lying well within the margins of the predicted mean and prevalence-functions. [N.B. These functional relations (Eq. 13) illustrated in Fig. 8b are useful for estimating an unknown SWB parameter *λ* in situations where the available data are incomplete, e.g*.* when only aggregated, community-level egg prevalence data are available instead of individual-level egg test data].

### Projecting the impact of MDA-based control

For this step, we applied our calibrated model to project MDA-control effects and post-treatment prevalence in one of the twelve Msambweni villages, Milalani, which was more intensively studied for the impact of MDA. The all-ages data used for analysis were collected in three village-wide surveys from 2000 (pretreatment baseline), 2003 (2 years post-treatment) and 2009 (six years post-treatment). The community-wide MDA in 2000 had a 79 % treatment coverage whereas the 2003 treatment coverage was only 41 %. The baseline (pretreatment) data served to calibrate the system, while the two post-treatment data sets were used to test model predictions. The coupled human-snail model consisted of two SWB groups (children, adults) linked to a single hypothetical snail site with a snail infection level approximating the average of five known Msambweni snail sites; SEI snail prevalence values in this experiment were taken as {x*, y*, z*} = {.63,.35,.02} based on field observations of bulinid snail PCR positivity for *S. haematobium* DNA [35] used to determine the number of ‘exposed’ snails and the observed annual frequencies of shedding snails [24, 36] for ‘infectious’ snails.

For our long term MDA prediction, we also scaled in overall population growth based on Kenyan demographics [37] and inter-seasonal variations of snail density over the 9-year study period. An ensemble of 150 likely calibrated parameter choices was drawn from the Milalani posterior and their 9-year histories computed. At each time, *t*, we took dynamic solutions and used equations (Eq. 13) to estimate the corresponding community egg-test results (prevalence and intensity) for child and adult age groups. We also ran this simulation for several values of the snail FOI -parameter rate, Λ_0_ (sporocyst establishment rate), ranging from 0.5 to 2 weeks.

**Fig. 10:**
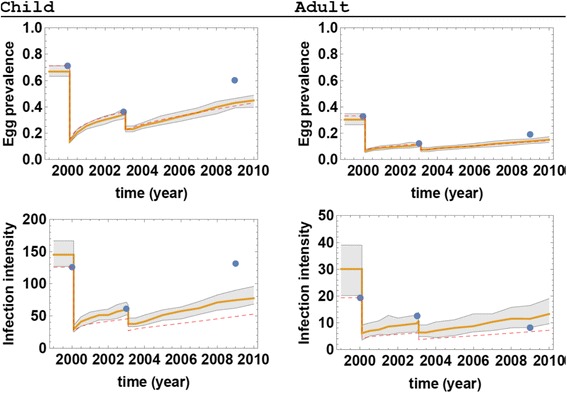
MDA control simulations for Milalani Village during the 2000–2009 period. The left panels represent children and the right panels represent adults. The ensemble prediction envelopes of prevalence (upper panels) and infection intensity (lower panels) based on current SWB simulations are shown in *gray* with their means represented by *yellow* lines. *Blue* circles represent observed field data. The dashed *red* line shows predicted control outcomes based on the earlier, simpler SWB model of [14]

The results shown in Fig. 10 correspond to 1/Λ_0_ = 1.5 week. We plotted the ensemble envelope along with predicted mean and compared to the three observed data points for prevalence and intensity, respectively. The data for 2000–2003 fell well within predicted margins. In modeling treatment outcomes after MDA in this test village, our prevalence and intensity estimates proved to be somewhat low by the last study year (Fig. 10). Infection intensity (measured by mean egg test) by year 9 is under-predicted for children and over-predicted for adults. This could be attributed to demographic and/or environmental changes that happened over 6-year intervening period, which were not accounted for in the model.

### Comparison between simple and advanced SWB models: parameter estimation and control predictions

The key for analysis and calibration of coupled human-snail systems are two forces of infection: snail-to-human *λ* and human-to-snail Λ. Both depend on snail/ human infectivity and the population densities of host and vector. For simple SI (susceptible-infected) snail system, *λ* is proportional to infected (patent) snail prevalence *λ* ∝*α y* with transmission coefficient *a* = “mean rate of worm accumulation in host” and infected (patent) snail prevalence *y*.

In the simple SWB system, snail FOI is taken proportional to mean human ‘infectivity’ = mean egg-release *E* by human hosts into the environment, Λ = *bE*, with transmission coefficient *b*. Transmission coefficients $\alpha$, *b* combine multiple factors and processes (population densities, human-snail contact rates, intermediate larval stages, etc.). Human infectivity function *E* depends on details of on worm aggregation and in-host biology.

For the earlier, simpler SWB, the calibration procedure [14, 15] employed algebraic relations between test data (prevalence *P*_*D*_ and mean intensity *E*_*D*_), model functions *P*_*W*_(*λ*) = 1 − *h*_0_(*λ*), and MWB $\overline{w}\left(\lambda \right)=\varDelta w{\displaystyle {\sum}_k{h}_k\left(\lambda \right)}$ expressed through dimensionless human FOI $\lambda =\frac{\alpha y}{\gamma \varDelta w}$. No distinction was made between worm and egg-test prevalence (*P*_*W*_ = *P*_*E*_) as egg-release was assumed to be proportional to MWB $\left(E=\rho \overline{w}\right)$ with fixed (uniform) fecundity/worm factor *ρ* to be estimated. That calibration proceeded in 2 steps: from prevalence equation *P*_*W*_(*λ*) = *P*_*D*_, we computed equilibrium λ^*^ then derived MWB w^*^ and the fecundity factor *ρ*

14 $$ {w}^{*}\approx \frac{\lambda^{*}\varLambda w}{\gamma +\mu };\kern1em \rho =\frac{E_D}{\phi \left(\overline{w}\right)} $$

with mating function *ϕ*(*w*) = *w*/2. The transmission coefficients were estimated as

15 $$ \alpha =\frac{\lambda \gamma\;\varDelta w}{y^{*}}\kern0.5em ,\kern0.5em b=\frac{v\;{y}^{*}}{E_D\left(1-{y}^{*}\right)} $$

This procedure could be extended to demographically and/or geographically linked SWB systems (see [14, 15]). No uncertainties entered the simple SWB (or its calibration), but explicit algebraic formulae allowed one to relate data noise to parameter estimation.

In comparing the predicted FOI curves (*P*_*E*_(*λ*, *ρ*_*B*_) and *P*_*W*_(*λ*)) for the two SWB systems (old and new), only the latter, *P*_*W*_, exists for the simple SWB, as no distinction was made there between worm-based and egg-test-based prevalence. For the new SWB, Fig. 8b shows quantile envelopes of *P*_*E*_ curves sampled over a range of biological parameters *ρ*_*B*_ (biological posterior). In general, we expect *P*_*E*_(*λ*) < *P*_*W*_(*λ*), as worm-carrying strata could contribute to zero egg count. For the simpler SWB, the red curve in Fig. 8b shows its significant departure from the median (and surrounding quantile envelopes) of the new SWB that incorporated host-worm biological interactions. We conclude that the old SWB significantly underestimated FOI λ^*^ and transmission rate *a* (Eq. 15), while overestimating fecundity factor *ρ* (Eq. 14).

To explore possible effect of such discrepancies on MDA control prediction, we took the above 9-year study for Milalani and compared simple SWB (dashed red line) with new calibrated envelope prediction in Fig. 10. Predicted prevalence of both models is reasonably close (within uncertainty envelopes), but the predicted infection intensity for children is underestimated using the simpler SWB (the dashed red line outside the newly calibrated envelope of likely values).

## Discussion

The newer calibrated SWB modeling approach appears well suited for simulating the effects of control interventions, particularly the effects of mass drug therapy. Other data inputs and other control strategies could easily be implemented within our setup, such as targeting treatment to specific populations, and modeling the efficiency of different post-treatment surveillance strategies. In the present analysis, we examined one case of long-term MDA outcomes in a Kenyan study site (population ~2,000) to validate our model in a dynamic setting and assess its predictive accuracy. Predicted relaxation patterns (means, envelopes, and quantiles) were found in good agreement with the observed data. Additional evidence comes from recent work [33] where our model and methodology was applied to program data from the Schistosomiasis Consortium for Operational Research and Evaluation (SCORE) on *S. haematobium* control from Mozambique. While this Mozambique data covered a wide spectrum of communities and control strategies, it was limited in terms of the number and age range of persons tested. To fill in some of the missing data gaps, we utilized certain parameter estimates from the Kenyan data studied here, assuming that similar population groups in both countries share comparable ‘biological’ infection parameters in terms of worm fecundity and density dependence. These calibrated inputs allowed us to build coupled SWB-snail systems for Mozambique communities and accurately simulate the MDA outcomes of the SCORE project.

Typical diagnostic tests for *Schistosoma* infection (based on egg counting in stool or urine) exhibit highly uneven distributions in host populations. These heterogeneities exist not only for broader communities, but for specific demographic groups that are assumed to be nearly the same in terms of risk. We conclude that several factors contribute to overdispersion of test results, among them uneven worm load due to varying exposure and/or host susceptibility [6], and irregular (clustered) egg release into human excreta [16]. Conventional population-based approaches to modeling (mean worm burden (MWB) models [8]) either ignore uneven burden (taking its population mean) or impose *ad-hoc* assumptions on worm distribution (e.g. the negative binomial [12]). Either approach has severe limitations (see [12]) that can reduce the utility of the model and the accuracy and robustness of its predictions. While autonomous agent, individual-based model simulations can allow for multiple heterogeneities [15], this alternative type of model has limited capacity in term of population size and program implementation on desktop/laptop platforms.

The SWB approach bridges the gap between these two types of models. It gives a consistent, assumption-free account of uneven worm load and naturally accommodates essential in-host biology, including worm mating probability and density-dependent reduction in fecundity. While the resulting SWB systems have more variables (depending on stratification), there are only a few model parameters per stratum that need to be calibrated, similar to the requirements for a low-dimensional MWB model. We note that promiscuous mating by female worms would enhance the continuation of transmission following MDA if, as generally seen, only partial elimination of worms is achieved with treatment. We have discussed the issue of mating patterns in some depth in our previous paper [12].

To better mimic the dynamics of human-to-snail-to-human parasite transmission, we have revisited and revised conventional approaches to modeling snail population and infection in our coupled SWB modeling systems. Typically, in MWB and related models, the parasite’s short-lived larval stages are not modeled but rather incorporated into an effective ‘force of infection’ (FOI) term, *λ*, for snail-to-human transmission, and Λ, for human-to-snail transmission. The latter, Λ, is often taken be proportional to human-to-snail infectivity in terms of cumulative *Schistosoma* egg release by the local human community, with a fraction of these eggs converting to miracidia to invade susceptible snails (see e.g. [9]). A new, closer look at the miracidium snail invasion process reveals a more likely nonlinear (saturated) form of snail FOI and sporocyst establishment [32]. The resulting estimates of human-to-snail transmission could provide an explanation for markedly different re-infection rates in some post-MDA communities [38] and the leveraged impact of human in-migration on persistence of transmission [39–41].

To better project future control program outcomes in terms of present-day data, we have developed a Bayesian calibration procedure for our model based on simulating the recognized imperfections of diagnosing infection based on egg-count data [17–22]. Other factors believed to be critical in accurate forecasting of *Schistosoma* prevalence include human age-group differences in exposure and susceptibility to infection [42–44], density-dependent (crowding) effects on egg production per worm [30, 45], limitations on mating success among adult schistosomes [4, 46], and the development of anti-fecundity immunity among older patients [47]. In our current SWB model, which included these factors [12], we found better calibration for the model in terms of projecting treatment and reinfection outcomes. The result of our calibration procedure is a posterior ensemble of likely SWB community values consistent with a given data set. The posterior distribution of parameter values for a given SWB community/group can be used to generate multiple community actualizations (based on (*λ*, *ρ*_*B*_) - choices) in order to simulate the range of likely outcomes. Each outcome is assigned a significance level determined by its likelihood weight. Thus any data/model uncertainties are propagated into “prediction uncertainty”. In most dynamic simulations, e.g. MDA control, these uncertainties can then be shown as prediction envelopes of possible outcomes.

We have applied the above calibration scheme to a specific data set for communities in coastal Kenya, where only *S. haematobium* is endemic. While the Kenyan communities differed markedly in terms of their risk and infection levels, we found their age-specific biological parameters *ρ*_*B*_ confined within the same close range, regardless of transmission intensity. This result supported our hypothesis on the constancy of in-host worm biology (mating, fecundity) and its parameterization. It was notable that the estimated crowding function (density-dependent fecundity) was different for child and adult groups, which appears to be in accordance with recent findings about the acquisition of anti-fecundity immunity in primates and humans [47]. Of note, our calibrated aggregation parameter *r* (Table 6) is also consistent with estimates of Hubbard et al. [16] for *Schistosoma japonicum* infection. A limitation of the present paper is its focus on *S. haematobium* (and its transmission features) in calibration of model predictions. However, work is in progress to repeat calibration and testing for *S. mansoni*-control projects in Kenya and Uganda, which should allow comparison of the estimated biological parameters for each species and help to determine if model recalibration is necessary for different species and for different ecological settings.

## Conclusions

The SWB provides an efficient, flexible, and viable approach for modeling *Schistosoma* transmission and control among stratified populations in simple and complex environments. SWB allows for inclusion of in-host biological factors and limitations of diagnostics, and is applicable to a broad range of treatment strategies. Most helpful to program managers, these new features allow us to predict diagnostic egg-test results for modeled SWB population subgroups and for communities at-large. Where only partial diagnostic data are available, the curves in Fig. 8 can serve to estimate parameters of transmission for program outcomes predictions.

This work is being extended to treatment projections for large-scale treatment trials currently implemented in both *S. mansoni*- and *S. haematobium-*endemic areas. For the near future, as part of the ongoing NTD Modelling Consortium project [48], our refined SWB model will be further validated against a new data and directly compared to the more traditional deterministic model of our consortium partners [49]. Fitted model predictions will be compared for likelihood and precision using two large data sets from recent control programs in sub-Saharan Africa: (i) the 2003–2006 *S. mansoni* data from the Ugandan National Schistosomiasis Control Programme in the African Great Lakes region, and (ii) 2010–2015 data from the SCORE/SCI multi-village operational research trial on *S. haematobium* control in Mozambique, evaluated in our previous paper [33]. Overall, we expect that the methodology developed in the current paper has a broad scope of applications for different *Schistosoma* species and more generally, for helminth/macroparasites infections where in-host biology plays an important role.

## Abbreviations

FOI, force of infection; MDA, mass drug administration; MWB, mean worm burden model; NB, negative binomial distribution; NTD, neglected tropical diseases; PDF, probability density function; SD, standard deviation of the mean; SWB, stratified worm burden model

## Additional files

Additional file 1: SWB codes15.nb [an example of the SWB model implemented using Mathematica v10.2 software (Wolfram Research, Champaign IL. USA)] (NB 35 kb) Additional file 2: Mixed SWB systems and equilibria (DOCX 90 kb) Additional file 3: Likelihood estimates for simulated egg test results (DOCX 204 kb) Additional file 4: Snail equilibria and calibration (DOCX 64 kb) Additional file 5: Infection data from Milalani village cross-sectional surveys (XLSX 55 kb)
