# Supplementary material for: Refined stratified-worm-burden models that incorporate specific biological features of human and snail hosts provide better estimates of Schistosoma diagnosis, transmission, and control
Source: Parasit Vectors. 2016 Aug 4;9:428. doi: 10.1186/s13071-016-1681-4 (PMC4973538; doi:10.1186/s13071-016-1681-4)
Supplement: Additional file 2: — Mixed SWB systems and equilibria (DOCX 90 kb) [file 13071_2016_1681_MOESM2_ESM.docx]

# Additional File 2: Mixed SWB systems and equilibria

Host population $H$ (community or subgroup) is divided into burden strata $\sum_{0}^{n} H_{k}=H$ based on their loads ($k\Delta w\leq w<(k+1)\Delta w$, for $H_{k}$), discretized with step$\Delta w$. Instead of population variables $H_{k}\left(t\right)$ we often use their fractions (prevalences) $h_{k}=H_{k}/H$. Transitions among strata $\left\{h_{k}\right\}$

are determined by human FOI $\lambda$ (= mean rate of worm accumulation divided by $\Delta w$), resolution rates $\gamma_{k}=k\gamma$ (due to natural worm mortality$\gamma$), population turnover $\mu$ (host mortality, aging, migration), and demographic source $\vec{S}=\left\{S_{k}\right\}$. Dynamic variables $\overrightarrow{h}\left(t\right)=\left\{h_{k}(t)\right\}$ obey a coupled differential system

$$\left\{\begin{matrix} \frac{dh_{0}}{dt}=-(\lambda+\mu)h_{0}+\gamma_{1}h_{1}+S_{0} \\ \vdots\\ \frac{dh_{i}}{dt}=\lambda h_{i-1}-(\lambda+\gamma_{i}+\mu)h_{i}+\gamma_{i+1}h_{i+1}+S_{i} \\ \vdots\\ \frac{dh_{n}}{dt}=\lambda h_{n-1}-(\gamma_{n}+\mu)h_{n}+S_{n} \end{matrix}\right.$$

which can be written in the matrix/vector form as

$\frac{d \vec{h}}{dt}=A\left(\lambda,\mu,\gamma\right)\cdot\vec{h}+\vec{S}$ ,

System has tri-diagonal transmission matrix

$$A\left(\lambda,\mu,\gamma\right)=\left[\begin{matrix} -(\lambda+\mu) & \gamma_{1} & & & \\ \lambda& \ddots& \ddots& & \\ & \lambda& -(\lambda+\gamma_{i}+\mu) & \gamma_{i+1} & \\ & & \ddots& \ddots& \gamma_{n} \\ & & & \lambda& -(\gamma_{n}+\mu) \end{matrix}\right]$$

Reduced MWB-type models can be derived from statistical moments of SWB .

For structured populations (demographically or geographically), each subgroup has its own SWB, with specific FOI$\lambda^{d}$, turnover$\mu^{d}$, and source term $\overrightarrow{S^{d}}$. A simple case is a child-adult system for vector variables ($\vec{h}_{C}; \vec{h}_{A}$ )

$$\left\{\begin{matrix} \frac{d}{dt}\mathop{h_{C}}\limits^{\to} =A\left(\lambda^{C},\mu^{C},\gamma\right)\cdot\mathop{h}\limits^{\to} _{C}+\mathop{S}\limits^{\to} _{C} \\ \frac{d}{dt}\mathop{h_{A}}\limits^{\to} =A\left(\lambda^{A},\mu^{A},\gamma\right)\cdot\mathop{h_{A}}\limits^{\to} +\mathop{S_{A}}\limits^{\to} \end{matrix}\right.$$

The children source $\overrightarrow{S^{C}}=\left\{b_{C},0,0,...\right\}$ coming from newborns ($b_{C}$= per capita birth rate = turnover $\mu^{C}$), enters the “infection free” $h_{0}$ -strata. The adult source is contributed by maturing children

$$\mathop{S^{A}}\limits^{\to} =\left\{S_{k}^{A}\right\}=\tau\frac{H_{C}}{H_{A}} \mathop{h^{C}}\limits^{\to}$$

Here $\left\{H_{C},H_{A}\right\}$ are relative populations of two groups, $\tau$ - maturation rate (=1/maturation age).

## SWB-equilibria

For stationary populations turnover rate is balanced by the cumulative source$\mu=\sum_{k=0}^{n} S_{k}$. Such systems have (endemic) equilibrium solutions. For a single SWB its equilibrium distribution depends on two dimensionless parameters $\left\{\frac{\lambda}{\gamma},\frac{\mu}{\gamma}\right\}$

For a coupled (child-adult) SWB we first solve for the child equilibrium

$$\vec{h}_{C}^{*}=\vec{h}_{C}^{*}\left(\frac{\lambda^{C}}{\gamma},\frac{\mu^{C}}{\gamma}\right)$$

then substitute $\vec{h}_{C}^{*}$ as the source term for adult equilibrium

$$\vec{h}_{A}^{*}=\vec{h}_{A}^{*}\left(\frac{\lambda^{A}}{\gamma},\frac{\mu^{A}}{\gamma},S_{A}\right)$$

Equilibrium equations - are consistently used in our calibration procedures.
