# Supplementary material for: Refined stratified-worm-burden models that incorporate specific biological features of human and snail hosts provide better estimates of Schistosoma diagnosis, transmission, and control
Source: Parasit Vectors. 2016 Aug 4;9:428. doi: 10.1186/s13071-016-1681-4 (PMC4973538; doi:10.1186/s13071-016-1681-4)
Supplement: Additional file 3: — Likelihood estimates for simulated egg test results (DOCX 204 kb) [file 13071_2016_1681_MOESM3_ESM.docx]

# Additional File 3: Likelihood estimates for the simulated egg test

The goal of our calibration scheme is to assign a likelihood weight to each parameter choice of the system (made up of a suite of biological and transmission parameters). The weights are based on estimated distance function between a simulated test and the real data.

Both data sets, real and simulated, are represented by their discretized (binned) distribution sequences $E_{S}=\left\{e_{0};e_{1};...;e_{20}\right\}$(simulated), $E_{D}=\left\{d_{0};d_{1};...\right\}$(real). A simulated test involves two random steps: i) randomly chosen hosts drawn from SWB strata with probabilities $\left\{h_{n}\right\}$, ii) random egg- release by each host determined by a hypothesized random value from an NB distribution of daily egg release. We think of $E_{D}$ as random realization of such process, so to compare $E_{S}$ with $E_{D}$ we generate for each parameters suite ($\lambda,p_{B}$), an ensemble $E\text{=}\left\{E_{S}\right\}$ (of several hundred simulated community egg-tests), and estimate the departure of the ensemble mean $\bar{E}$ from $E_{D}$ relative to ensemble covariance $\sigma$.

The truncated covariance matrix $\sigma$ of test ensemble $E$ is represented by the first few principle components, corresponding to its largest eigenvalues: $\lambda_{1}>\lambda_{2}>...>\lambda_{m}$ , truncated at $\lambda_{m}\geq\theta\lambda_{1}$with the truncation threshold $0<\theta<1$, knowing our calibration scheme could be sensitive to the choice of $\theta$. The corresponding “likelihood distance” between $E$ and $E_{D}$ is defined as

$$d\left(E,E_{D}\right)=\left(\bar{E}-E_{D}\right)\cdot\sigma^{-1}\cdot\left(\bar{E}-E_{D}\right)$$

with quasi-inverse matrix $\sigma^{-1}$. In Bayesian terminology, the likelihood estimate can be viewed as a conditional probability$P\left(E_{D}|E\right)$. For normal distributions, likelihood estimates depend on a suitable mean-square distance function. Assuming $E_{S}$ is approximately normal, we estimate the distance (error) function, $d\left(E_{S},E_{D}\right)$ , using principal component analysis of the covariance matrix $\sigma^{-1}$of $E_{S}$.

Distance gives rise to likelihood weights for any parameter $\vec{p}=\left(\lambda,p_{B}\right)$

Likelihood weights $\left\{W\left(\lambda,p_{B}\right)\right\}$ define a posterior empirical distribution $D=D\left(\lambda,p_{B}\right)$ on the $\left(\lambda,p_{B}\right)$ -space.

For each of multiple random choices $\left(\lambda,p_{B}\right)$ from $D$, one can assign the corresponding likelihood $W\left(\lambda,p_{B}\right)$. The weighted distribution defined by the random selections and the likelihoods could be the second posterior distribution $D_{2}$. There is a minor difference between $D$ and $D_{2}$ specified by the scalar (12 for $D$ and 12/2=6 for $D_{2}$).

## Sensitivity of our calibration scheme

The threshold of principal component truncation, $\theta$ , determines how many components are to be used in the approximation of $\sigma^{-1}$ (typically 10-12 with $\theta$=0.001 and 5-12 with $\theta$=0.1, when 12 is the number of total components). (Figure 1, below)

| a. | b. |
| --- | --- |
| 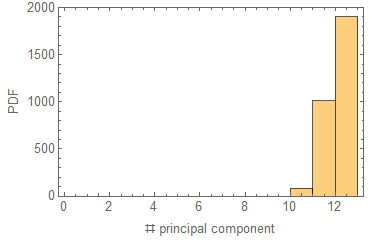 | 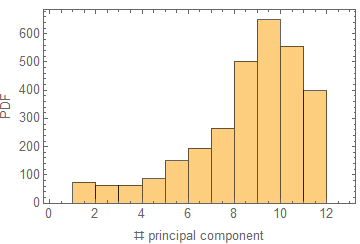 |

Figure . Distribution of the number or principal components for an ensemble set consists of the 3000 $\left(\lambda,p_{B}\right)$ choices with $\theta$=0.001 (a) and $\theta$=0. 1 (b)

Although we used a fixed $\theta$=0.001 for our calibrations of Msambweni villages, our calibration scheme could be sensitive to the choice of the parameter. (Figure 2)

| a. | b. |
| --- | --- |
| 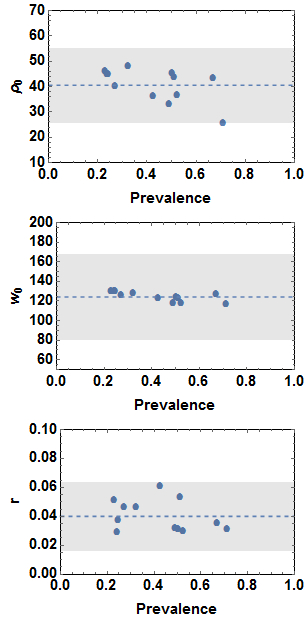 | 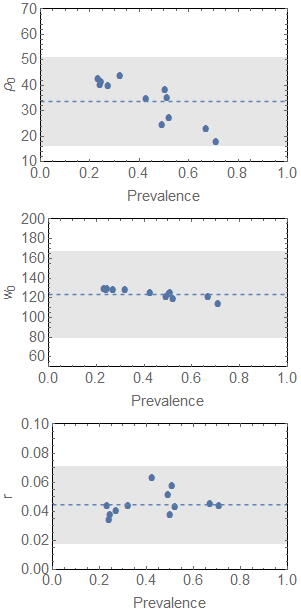 |

Figure . Estimated biological parameters (ensemble means) for children in twelve Msambweni villages plotted against field data (egg-prevalence) with (a) $\theta$=0.001 (same as Figure 6(a) in the main text) and (b) $\theta$=0. 1
