# Supplementary material for: Refined stratified-worm-burden models that incorporate specific biological features of human and snail hosts provide better estimates of Schistosoma diagnosis, transmission, and control
Source: Parasit Vectors. 2016 Aug 4;9:428. doi: 10.1186/s13071-016-1681-4 (PMC4973538; doi:10.1186/s13071-016-1681-4)
Supplement: Additional file 4: — Snail equilibria and calibration (DOCX 64 kb) [file 13071_2016_1681_MOESM4_ESM.docx]

# Additional File 4: Snail equilibria and calibration

SEI snail model has 3 dynamic variables (snail densities) *x*- susceptible, *y*- pre-patent, *z*-patent, $N=x+y+z$ - total, related via FOI $\Lambda$ , recovery rate *r* (prepatency period = 1/*r*), patency conversion fraction *c*, source (reproduction/growth) term $\beta$ and snail mortality $\nu$

$$\to^{\beta} \mathop{x}\limits_{\begin{matrix} \downarrow\\ \nu\end{matrix}} \rightleftarrows_{\left(1-c\right)r}^{\Lambda} \mathop{y}\limits_{\begin{matrix} \downarrow\\ \nu\end{matrix}} \to^{c r} \mathop{z}\limits_{\begin{matrix} \downarrow\\ \nu\end{matrix}}$$

They obey differential equations

$$\begin{matrix} \frac{dx}{dt}=\beta-\Lambda x-\nu x+r\left(1-c\right)y \\ \frac{dy}{dt}=\Lambda x-\left(r+\nu\right) y \\ \frac{dz}{dt}=c r y-\nu z \end{matrix}$$

We assume population dynamics driven by logistic source with maximal reproduction rate $\beta_{0}$and carrying capacity *K*,

$\beta=\beta_{0}\left(1-N/K\right)\left(x+y\right)$,

-only susceptible and prepatent populations can reproduce. In stationary transmission environment, i.e. fixed parameters ($K,\beta_{0},\Lambda$) and FOI $\Lambda$, system has equilibrium solution

$N^{*}=K\left[1-\frac{\nu}{\beta_{0}}-\frac{r c \Lambda}{\beta_{0}\left(\Lambda+r+\nu\right)}\right]$-total population,

and prevalences

$$\begin{matrix} x^{*}/N^{*}=\frac{\nu\left(\nu+r\right)}{\left(c r +\nu\right)\Lambda+\nu\left(\nu+r\right)} \\ y^{*}/N^{*}=\frac{\nu\Lambda}{\left(c r +\nu\right)\Lambda+\nu\left(\nu+r\right)} \\ z^{*}/N^{*}=\frac{c r \Lambda}{\left(c r +\nu\right)\Lambda+\nu\left(\nu+r\right)} \end{matrix}$$

expressed through $\left(\nu,c,r\right)$ and FOI $\Lambda$. Equation constraints biological and transmission parameters, namely $N^{*}>0$ implies $\beta_{0}-\nu>c r$. Some parameters of system are known, e.g. snail mortality $\nu$ , patency recovery rate *r*, conversion fraction ($.05<c<.15$).

The most important for analysis of coupled human-snail systems is FOI $\Lambda$- a nonlinear function of the product $B H E$ (mean host infectivity *E*, population size *H,* human-to-snail transmission rate *B*)

$$\Lambda=\Lambda_{0}\left(1-\exp\left[-B H E\right]\right)$$

Combining equations - with the available human-snail infection data allows us to estimate FOI $\Lambda$ and transmission coefficients$A,B$(snail-to-human and human-to-snail).
